# Silibinin and Naringenin against Bisphenol A-Induced Neurotoxicity in Zebrafish Model—Potential Flavonoid Molecules for New Drug Design, Development, and Therapy for Neurological Disorders

**DOI:** 10.3390/molecules27082572

**Published:** 2022-04-15

**Authors:** Geethanjali Thayumanavan, Srikanth Jeyabalan, Shivkanya Fuloria, Mahendran Sekar, Monica Ravi, Logesh Kumar Selvaraj, Logeshwari Bala, Kumarappan Chidambaram, Siew Hua Gan, Nur Najihah Izzati Mat Rani, M. Yasmin Begum, Vetriselvan Subramaniyan, Kathiresan V. Sathasivam, Dhanalekshmi U. Meenakshi, Neeraj Kumar Fuloria

**Affiliations:** 1 Department of Pharmacology, Sri Ramachandra Faculty of Pharmacy, Sri Ramachandra Institute of Higher Education and Research (DU), Porur, Chennai 600116, Tamil Nadu, India; geethathayumanavan14@gmail.com (G.T.); srikanth.j@sriramachandra.edu.in (S.J.); monicaravi2898@gmail.com (M.R.); logezh@gmail.com (L.K.S.); logeshwarib@sriramachandra.edu.in (L.B.); 2 Faculty of Pharmacy, AIMST University, Bedong 08100, Kedah, Malaysia; 3 Department of Pharmaceutical Chemistry, Faculty of Pharmacy and Health Sciences, Royal College of Medicine Perak, Universiti Kuala Lumpur, Ipoh 30450, Perak, Malaysia; mahendransekar@unikl.edu.my; 4 Department of Pharmacology, College of Pharmacy, King Khalid University, Abha 62529, Saudi Arabia; kumarappan@kku.edu.sa; 5 School of Pharmacy, Monash University Malaysia, Bandar Sunway, Subang Jaya 47500, Selangor, Malaysia; gan.siewhua@monash.edu; 6 Faculty of Pharmacy and Health Sciences, Royal College of Medicine Perak, Universiti Kuala Lumpur, Ipoh 30450, Perak, Malaysia; najihah.izzti@gmail.com; 7 Department of Pharmaceutics, College of Pharmacy, King Khalid University, Abha 61421, Saudi Arabia; ybajen@kku.edu.sa; 8 Faculty of Medicine, Bioscience and Nursing, MAHSA University, Jalan SP 2, Bandar Saujana Putra, Jenjarom 42610, Selangor, Malaysia; drvetriselvan@mahsa.edu.my; 9 Faculty of Applied Sciences, AIMST University, Bedong 08100, Kedah, Malaysia; skathir@aimst.edu.my; 10 College of Pharmacy, National University of Science and Technology, Muscat 130, Oman; dhanalekshmi@nu.edu.om; 11 Center for Transdisciplinary Research, Department of Pharmacology, Saveetha Institute of Medical and Technical Sciences, Saveetha Dental College and Hospital, Saveetha University, Chennai 600077, Tamil Nadu, India

**Keywords:** Bisphenol A, zebrafish, neurotoxicity, silibinin, naringenin, neuroprotective

## Abstract

Bisphenol A (BPA), a well-known xenoestrogen, is commonly utilised in the production of polycarbonate plastics. Based on the existing evidence, BPA is known to induce neurotoxicity and behavioural issues. Flavonoids such as silibinin and naringenin have been shown to have biological activity against a variety of illnesses. The current research evaluates the neuropharmacological effects of silibinin and naringenin in a zebrafish model against neurotoxicity and oxidative stress caused by Bisphenol A. In this study, a novel tank diving test (NTDT) and light–dark preference test (LDPT) were used in neurobehavioural investigations. The experimental protocol was planned to last 21 days. The neuroprotective effects of silibinin (10 μM) and naringenin (10 μM) in zebrafish (Danio rerio) induced by BPA (17.52 μM) were investigated. In the brine shrimp lethality assay, the 50% fatal concentrations (LC_50_) were 34.10 μg/mL (silibinin) and 91.33 μg/mL (naringenin) compared to the standard potassium dichromate (13.15 μg/mL). The acute toxicity investigation found no mortality or visible abnormalities in the silibinin- and naringenin-treated groups (LC_50_ > 100 mg/L). The altered scototaxis behaviour in LDPT caused by BPA was reversed by co-supplementation with silibinin and naringenin, as shown by decreases in the number of transitions to the light zone and the duration spent in the light zone. Our findings point to BPA’s neurotoxic potential in causing altered scototaxis and bottom-dwelling behaviour in zebrafish, as well as the usage of silibinin and naringenin as potential neuroprotectants.

## 1. Introduction

Flavonoids are a type of naturally occurring polyphenolic compound present in a wide variety of plants, including fruits, vegetables, cereals, teas, and wines, and they play a significant role in the diet. Flavonoids have numerous biological functions, including neuroprotective effects [1] caused by neurotransmitter regulation and stimulation of hippocampal neurogenesis [2]. Furthermore, dietary flavonoids have been shown to improve memory and learning via neuronal signaling pathways, while also slowing the progression of debilitating brain illnesses such as Parkinson’s and Alzheimer’s disease [3].

Milk thistle (*Silybum marianum*) has been used for neuroprotection, liver detoxification, and the treatment of bile duct and gallbladder diseases [4,5]. Silymarin, a lipophilic extract derived from the seeds (fruit) of milk thistle, contains a complex of flavonols derived from lignan, including silibinin (silybin), silydianin, silychristin, and isosilybin. However, silibinin, the main bioactive component of the extract, accounts for 50–70% of the extract. Silibinin concentrations in typical pharmaceutical products range from 20 to 40% [6]. Silibinin, in addition to its hepatoprotective properties, is rich in antioxidants and impacts a variety of cell-signaling pathways, resulting in a decrease in pro-inflammatory mediators [7]. Currently, silibinin is being studied as a possible anticancer and chemopreventive drug [8].

Naringenin (4′,5,7-trihydroxy flavanone-7-rhamnoglucoside) is a bioflavonoid that can be found in many different fruits and vegetables, such as grapes and citrus. The sourness and bitterness of a fruit are caused by this component [9]. According to research, naringenin can help people with cognitive problems by cutting down on oxidative stress, neuroinflammation, and neuroapoptotic pathways [10,11,12].

**Figure 1:**
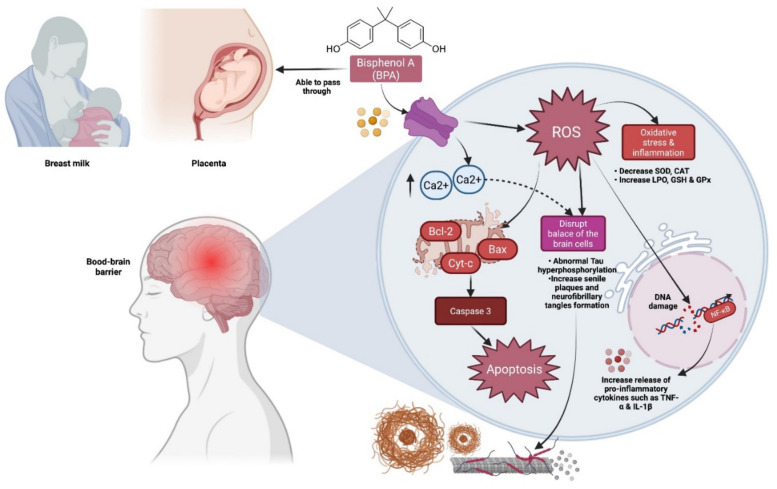
Cognitive impairments caused by BPA have a specific mechanism. BPA can travel past the placenta, the blood–brain barrier, and into breast milk, where it can be passed on to neonates. BPA also elevates reactive oxygen species (ROS) and intracellular calcium (Ca^2+^), which can lead to hyperphosphorylation of Tau, neurofibrillary tangles, and senile plaques development, resulting in dementia or Alzheimer’s disease (AD). Additionally, BPA has been linked to increases in oxidative stress, inflammation, and apoptosis in cells.

Bisphenol A (BPA) is an anthropogenic xenoestrogen that has been found to be an endocrine disruptor that is acutely hazardous to freshwater and marine animals in concentrations ranging from 1000 to 10,000 g/L. BPA is widely present in the environment, having been found in dust and even human urine [13]. Due to its lipophilic nature, BPA can pass through the placenta and the blood–brain barrier, as well as being passed on to neonates through breast milk. Furthermore, most research indicates that BPA promotes oxidative stress, emotional disturbances, cognitive impairment, carcinogenicity, and inflammation. Furthermore, oxidative stress has been associated with a variety of health problems, including ageing, cardiovascular disease, neurodegeneration, and inflammation [14] (Figure 1). Several previous studies have shown that increases in reactive oxygen species (ROS) generation fuel BPA’s hazardous potential. The removal of BPA from bodies of water near human-occupied areas poses a severe concern, making the aquatic environment a vulnerable target for sewage treatment via waste outflow and natural corruption.

The zebrafish (*Danio rerio*) has emerged as a significant animal model for a broad range of human brain disorders, including Alzheimer’s disease and Parkinson’s disease. [15,16]. Approximately 82% disease-related genes have orthologues in the completely sequenced zebrafish genome [17]. Zebrafish brain atlases and gene expression datasets can be utilised to explore the genetic and neuroanatomical aspects of brain areas connected to neuropsychiatric diseases. It is possible to find new therapeutic targets and their molecular links by simulating human brain conditions in zebrafish [18]. Preclinical in vivo models of both the larval stage and adult zebrafish can be manipulated genetically, pharmacologically, and experimentally. As zebrafish embryos and larvae are transparent and tiny, they are ideal for imaging the activity in the brain, optical manipulation, and high-throughput screenings of molecular targeted therapies and candidate genes [19].

As a trademark example of social reactions [20] to multiple harmful synthetic compounds and stress situations, including those of therapeutic interventions, the zebrafish is currently considered an appropriate animal model of the aquatic environment in various preclinical investigations, being similar to mammals. Natural chemical intervention as a prophylactic or therapeutic strategy to prevent BPA-induced neurotoxicity is a promising option in this regard. Several studies in animal models and humans have indicated that natural compounds with antioxidant properties can help halt the progression of neurodegenerative illnesses.

To date, rampant use and unregulated dumping of plastics pose risks to the atmosphere. BPA levels in water bodies near human settlements have risen as a result of the widespread use and disposal of BPA-containing products, which poses a risk to human health. Neurodegenerative disorders such as Alzheimer’s and Parkinson’s disease pose risks to future generations. BPA is neurotoxic and has a deleterious impact on the evolution of serious neurological disorders [21]. Because of its lipophilic nature, BPA is able to pass through the blood–brain barrier and cause neurotoxicity in the brain. There must be an equilibrium between the production of free radicals and the antioxidant defence system to ensure that ROS are not overproduced and that intracellular signalling pathways are not disrupted [22]. Few animal studies have reported the effects of BPA in the neurological systems. BPA can cause anxiety-like behaviour, memory impairment, sexual differences, hyperactivity, and changes in levels of nitrox oxide production [23,24,25,26,27]. A study revealed that prenatal and neonatal chronic exposure to BPA induces memory impairment and is associated with a reduction in the acetylcholine and decreased hippocampal cholinergic transmissions [28]. These effects may be due to increases in cholinesterase enzymes, which are potential targets for neurodegenerative diseases.

Generally, oxidative stress is cited as the fundamental cause of a variety of health conditions, including neurodegenerative diseases [29]. In such cases, an aquatic model such as the zebrafish may be most suited for studying the influence of BPA on neurobehavioural responses, antioxidant levels, and neuromorphology, using potent flavonoids such as silibinin and naringenin as a possible neuroprotective approach. Nonetheless, research on the neuroprotective effects of silibinin and naringenin against BPA-induced neurotoxicity, which might alter neurobehavioural responses and oxidative stress, is limited [30]. Hence, the present study aimed to assess the neuropharmacological effects of silibinin and naringenin in a zebrafish model against BPA-induced neurotoxicity and oxidative stress.

## 2. Materials and Methods

### 2.1. Chemicals

Flavonoids (silibinin and naringenin) and other chemicals of analytical grade were obtained from Thamil Kumaran Enterprises, Chennai, Tamilnadu, India. Adult wild-type zebrafish were purchased from a local aquarium shop in Kolathur, Chennai, Tamilnadu, India.

### 2.2. Brine Shrimp Lethality Assay

*Artemia salina,* a zooplankton also known as brine shrimp, is used to feed larval fish in the Artemia genus of aquatic crustaceans. Brine shrimp cysts (150 mg) were incubated for hatching in a conical container (separating funnel) filled with sea water. A 0.06% yeast solution was added to the hatching chamber after 24 h to feed the larvae, which were then placed in seawater and aerated for 48 h to keep them alive. The nauplii collected from the hatching chamber after 48 h were free of egg shells. When the nauplii were 48 h old they had reached the II-III larval stages, after which they were employed in the testing.

Using a Pasteur pipette, 10–15 nauplii were removed from the hatching chamber and were placed in the 24-well plates. The procedure was performed using a Pasteur pipette and a microscope. During the larval passage, a volume of no more than 1 mL should be transferred to avoid affecting the overall volume of the test system [31]. Silibinin and naringenin, as well as a positive control (potassium dichromate), were prepared in various concentrations (0.1, 1.0, 10.0, 100.0, and 1000.0 µg/mL) and 0.5 mL was added to each well containing sea water and kept at room temperature for 24 h to allow adequate contact with active nauplii in the well plates. After 24 h, the number of surviving nauplii in each well was counted. The percentage of larvae that died in the test and control systems was determined by comparing the mean survival rate of the two systems. The best-fit line plotting concentration versus lethality yielded the values for a lethal concentration of 50% (LC_50_) [32].

Toxicity criteria for fractions were defined as LC_50_ values >1000 μg/mL (nontoxic), ≥ 500 ≤ 1000 μg/mL (weak toxicity), and <500 μg/mL (toxic), as calculated below:

(1) $$\%\;\mathrm{Death}=\frac{\mathrm{the}\;\mathrm{number}\;\mathrm{of}\;\mathrm{dead}\;\mathrm{nauplii}}{\mathrm{the}\;\mathrm{number}\;\mathrm{of}\;\mathrm{dead}\;\mathrm{nauplii}\;+\;\mathrm{the}\;\mathrm{number}\;\mathrm{of}\;\mathrm{live}\;\mathrm{nauplii}}$$

### 2.3. Acute Toxicity Test 

The OECD 203 guideline for fish acute toxicity experiments and the methodology for determining the chemical concentration required 50% of the fish to be killed [33]. In accordance with the guidelines, the fish were exposed to the test substance for 96 h. At 24, 48, 72, and 96 h, the fish mortality rates were recorded and the LC_50_ values were determined (Table 1). Briefly, zebrafish were procured and acclimated for seven days to the laboratory conditions. The temperatures were maintained at 21–25 °C for 12–16 h during the photoperiod. The fish were fed twice a day for 24 h prior to the experiment. After this, for every 24 h, the dissolved oxygen, pH, and temperature were measured. To confirm that the LC_50_ was greater than the concentration, a limit test was conducted at 100 mg/L (active ingredient). For the acute toxicity study, ten fish per test concentration of 100 mg/L and a control group with reverse osmosis were used. The test system was kept static throughout the observation time as part of a static approach (96 h). If there was no apparent movement after 24, 48, 72, or 96 h, the fish were deemed dead. During the observation period, visible abnormalities such as (1) loss of equilibrium, (2) swimming behaviour, (3) respiratory function, (4) pigmentation, and (5) other clinical symptoms were recorded to determine the signs of toxicity and the LC_50_ values were determined based on the appropriate exposure times.

### 2.4. Neuropharmacological Evaluation

In order to reduce the crowding stress, the fish were kept in 3 L of water. The procedure was conducted without using the drug in the control group. All solutions were made fresh daily. On each day of treatment, fresh Bisphenol-A and flavonoid solutions (silibinin and naringenin) were made. The treatment period lasted 21 days, while the major assessment of behavioural parameters was performed on day 22 (Table 2).

#### 2.4.1. Novel Tank Diving Test (NTDT)

The NTDT is commonly used to study zebrafish exploratory behaviour [34]. Initially the fish tend to sink to the bottom of the new tank and subsequently swim up to the higher sections, so this challenge is based on that behaviour [35]. This archetypal behaviour test was based on the fact that zebrafish spent the majority of their time in the bottom zone of a novel dive tank (“bottom-dwelling”). NTDT was utilised to study the exploratory behaviour of the zebrafish in the ANY maze video tracking system. Evaluations were performed on positions along the wall vs. the centre of a rodent field (lower, middle, and upper levels) [36]. The zebrafish’s inherent inclination to seek protection in an unknown place was exploited by the innovative tank diving test, which was essentially similar to the mouse open field test.

The irregular movements, freezing, latency, and transitions into the upper parts of the tank can be measured and compared by researchers. These metrics can be used to compare fishes in different tanks. As part of the experiment, the fish were relocated from their home tank to a clean observation tank with water that had never been exposed to other fish. The tanks were divided into three equal virtual horizontal sections labelled on the outer walls of the apparatus, which was thin, transparent, and designed for little lateral movement but easy vertical and horizontal movements. A white, opaque self-adhesive plastic film was applied to both the lateral and backsides to reduce the impact of the surrounding area and to ease observation. Aside from this, a number of metrics were tracked, such as how long it took to get to the tank’s uppermost portion and the latency to reach the upper part of the tank.

#### 2.4.2. Light–Dark Test

Regarding scototaxis (desire for darkness), zebrafish were found to prefer darker settings using the LDPT [37]. While the novelty of the environment is the primary aversive stimulus in the novel tank diving test, in the scototaxis test the conflict between the motivations for approach and avoidance is the primary driving force [38]. The test is deceptively simple, depending on the fish’s exploration in a black and white tank to show their preference, similar to the mouse’s light–dark box. To conduct the light–dark test, we used a glass tank that measured 18 × 9 × 7 cm in length, width, and height, respectfully. A black chart was used to divide the tank into two equal-sized dark and white sections. The water level in the tank was raised to 4 cm (about 3 L) to allow the zebrafish to freely swim from one side of the tank to the other. ANYmaze was used to perform the data analysis by analysing the behaviour via its tracking systems.

### 2.5. In Silico Docking Analysis

#### Identification of Ligand and Protein

The in silico docking investigation was carried out using the Molegro Virtual Docker. The Protein Data Bank was used to collect the proteins for docking experiments in PDB format. The flavonoids silibinin and naringenin were retrieved from the pubChem chemical database and stored in mol format for docking investigations. The 3D structure of the target protein disease was obtained from the RCSB Protein Data Bank [39]. The Protein Data Bank was used to obtain the X-ray crystal co-ordinates for AChE (PDB ID: 1B41) and BChE (PDB ID: 4BDS). The standard drugs donepezil and rivastigmine were also obtained from the DrugBank database and saved in mol format.

For AChE (PBD: 1B41), donepezil was used as a standard, while for BChE, rivastigmine was used as a standard (PBD: 4BDS). The ligands, including silibinin, naringenin, and standard drugs, were imported into the Molegro virtual docker (MVD) workspace and prepared for docking. Both silibinin and naringenin docking scores and patterns were compared to those of standard drugs [40].

## 3. Results

### 3.1. Brine Shrimp Lethality Assay

**Figure 2:**
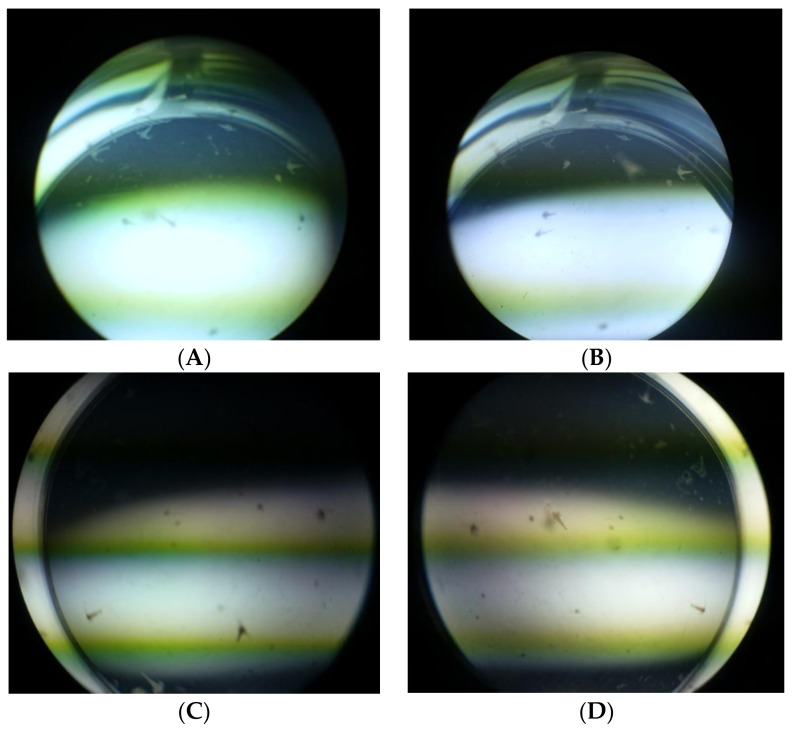
Brine shrimp lethality at 24 h in a 24-well plate: (**A**) potassium dichromate (1000 µg/mL); (**B**) naringenin (1000 µg/mL); (**C**) silibinin (1000 µg/mL); (**D**) control.

**Figure 3:**
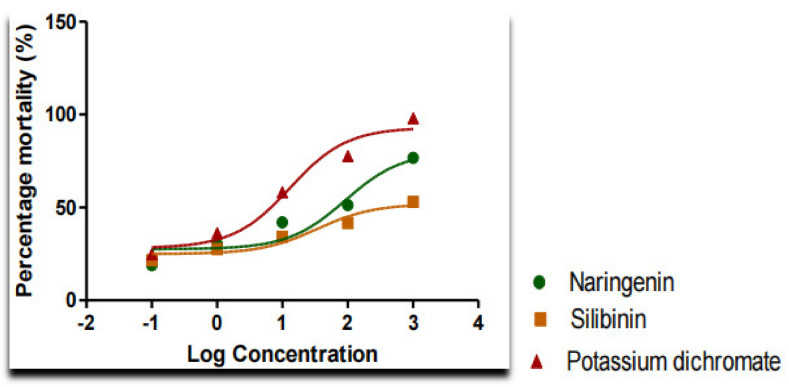
Brine shrimp lethality assay of the treated compounds.

The brine shrimp lethality bioassay can be used as a convenient monitoring tool for screening and fractionation in the discovery and monitoring of bioactive natural compounds rather than the more time-consuming and expensive in vitro anticancer tests. According to the 24 h assay, nearly all shrimp in the control sets survived the study. The shrimp began to die after 12 h at the highest treatment concentration (1000 µg/mL) and they were completely dead after 21 h (Figure 2). The potassium di-chromate positive control, which had a cytotoxic LC50 value of 13.15 µg/mL, resulted in the complete death of the shrimps. Silibinin and naringenin, after 24 h, were found to be toxic to the brine shrimp at LC50 values of 34.10 µg/mL and 91.33 µg/mL, respectively (Table 3 and Figure 3).

The effectiveness of the bioassay of *Artemia salina* L. (*Artemiidae*) for predicting the toxicity of plant extracts was evaluated by comparing it with the LD_50_ value results obtained from the acute toxicity tests in zebrafish, thereby providing a correlation between the brine shrimp lethality assay and the acute toxicity test. After 24 h of exposure to the compounds, the LC_50_ was found to be <1000 μg/mL. In the toxicity evaluation of plant extracts using the brine shrimp bioassay, an LC_50_ value of 1000 μg/mL was deemed bioactive. The flavonoids silibinin and naringenin had LC_50_ values of less than 1000 μg/mL in this investigation, clearly indicating their biological activity (Table 4).

### 3.2. Acute Toxicity Test 

The limit tests of silibinin and naringenin were performed in accordance with the OECD 203 guidelines to demonstrate that the LC_50_ was greater than the 100 mg/L concentration. During the test’s observation period (i.e., at 24, 48, 72, and 96 h), no visible abnormalities such as (1) loss of equilibrium, (2) swimming behaviour, (3) respiratory function, (4) pigmentation, or (5) other clinical signs and no mortality were observed in the silibinin- and naringenin-exposed and control groups (Table 5). The fish exposed to 100 mg/L flavonoids exhibited normal behaviour and swimming patterns with no mortality. After 72 h, the colour of the naringenin solution changed, which may be attributed to the physicochemical properties of the naringenin, since no mortality was observed in the naringenin-treated group.

The guideline’s threshold approach tackles fish toxicity by first adopting a single concentration test (limit test) as the threshold concentration (TC), which requires fewer fish than the whole acute fish toxicity test. If the TC is greater than 100 mg/L, the test substance concentration in the limit test should be 100 mg/L. The absence of mortality following a short-term exposure implies that the fish are not the most sensitive group of test organisms and that the LC50 is greater than the threshold concentration with at least 99% confidence. It is important to keep track of any sub-lethal effects. In addition, the test should be stopped when one of the test group’s fish dies or becomes moribund, which necessitates a thorough investigation. According to the OECD TG 203 limit test, no acute toxicity effects were related to silibinin and naringenin at the investigated concentrations in the acute toxicity test and the LC50 was >100 mg/L.

### 3.3. Neuropharmacological Evaluation in Zebrafish

The behavioural parameters were assessed using a novel tank and a light–dark test after a 21 day treatment period.

#### 3.3.1. Novel Tank Test: Silibinin and Naringenin Restores the Bottom Dwelling and Explorative Behaviour of Zebrafish following Co-Supplementation with BPA

**Figure 4:**
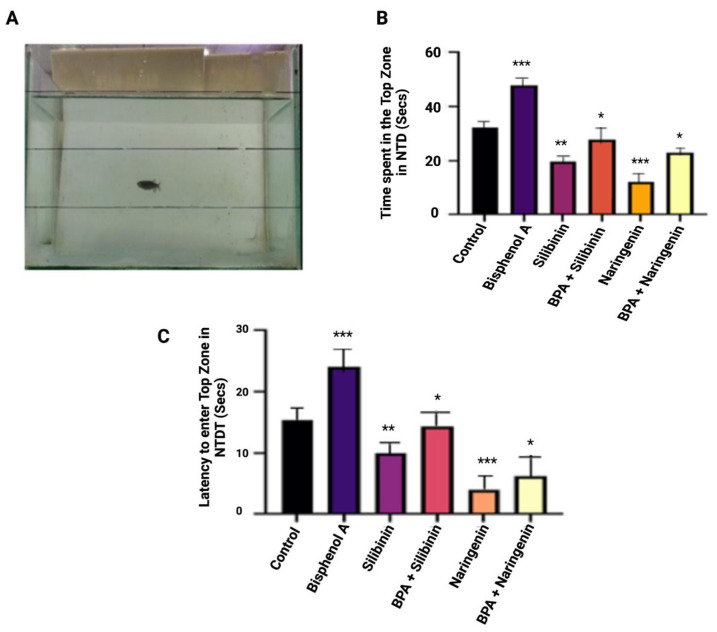
(**A**) Novel tank set up and (**B**) time spent in the top zone in the NTD (**C**) Latency to enter the top zone in NTDT. The values are expressed as means ± SEM, *n* = 10 fish in each groups. The significance was analysed using a one-way ANOVA. Note: * *p* < 0.05, ** *p* < 0.01, *** *p* < 0.001 compared with the control group.

In NTDT, BPA-exposed groups spent more time in the top zone as compared to control groups, suggesting that the duration spent in the top zone increased. In the BPA-exposed group, the delay in the top zone entry was much lower than that in the control group. When compared to the BPA-exposed group, co-supplementation of silibinin and naringenin significantly decreased the BPA-induced modification in the time spent in the top zone and the latency in entering the top zone, indicating a neuroprotective effect. The findings of this study clearly indicate that silibinin and naringenin have potential neuroprotection effects against BPA-induced neurobehavioural abnormalities (Figure 4).

#### 3.3.2. Light/Dark Test

**Figure 5:**
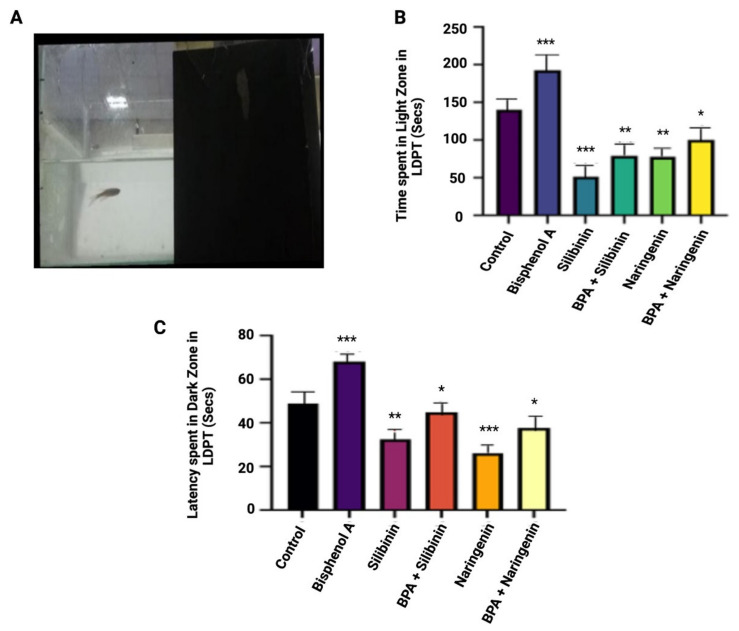
(**A**) Light and dark preference tank test and (**B**) times spent in the light cone in LDPT (Seconds) (**C**) The latency spent in the dark zone in LDPT (seconds). The values are expressed as means ± SEM, *n* = 10 fish in each groups. Significance was determined using a one-way ANOVA. Note: * *p* < 0.05, ** *p* < 0.01, *** *p* < 0.001 compared with the control group.

During the light and dark tests, the control group spent 137 s in the light compartment, which was separated into light and dark zones. In the light compartment, all control fish lasted fewer than 150 s. Following chronic waterborne BPA exposure and flavonoid co-supplementation, silibinin and naringenin treatment groups lasted for 130 s and 151 s, respectively, during the latency to reach the dark zone test in a 5-min session in LDPT. In comparison to the control, chronic waterborne exposure to BPA significantly affected the scototaxis behaviour of zebrafish, as evidenced by the increased transition to the light zone and the duration spent in the light zone. In LDPT, BPA exposure resulted in a significant increase in the latency to enter into the dark zone as compared to the control groups (Figure 5). In comparison to the BPA group, silibinin and naringenin significantly improved the altered scototaxis behaviour of zebrafish in the BPA + silibinin and BPA+ naringenin groups.

### 3.4. In Silico Docking Analysis

The docking of flavonoids with cholinesterase receptors such as AChE (1B41) and BChE (4BDS) revealed the highest affinity when compared to the standard drugs of the respective targets, as evidenced by the MolDcok score and rerank score, as well as the H bond energy. Docking was done using a grid resolution of 0.30 A^o^ and a maximum of 1500 iterations on a single population of 50 individuals for each of the 10 independent runs. The active binding site was regarded as a rigid molecule, while the ligands were treated as flexible molecules, allowing for all non-ring torsions. In silico investigations revealed that silibinin has the highest binding affinity for AchE, followed by BChE. In addition, prior in vitro research revealed that silibinin inhibits p38 MAPK in neurodegenerative illnesses such as Parkinson’s and Alzheimer’s disease [41].

**Figure 6:**
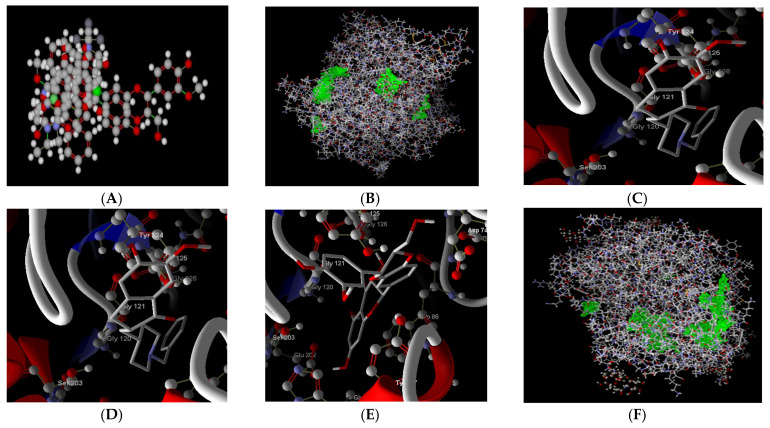

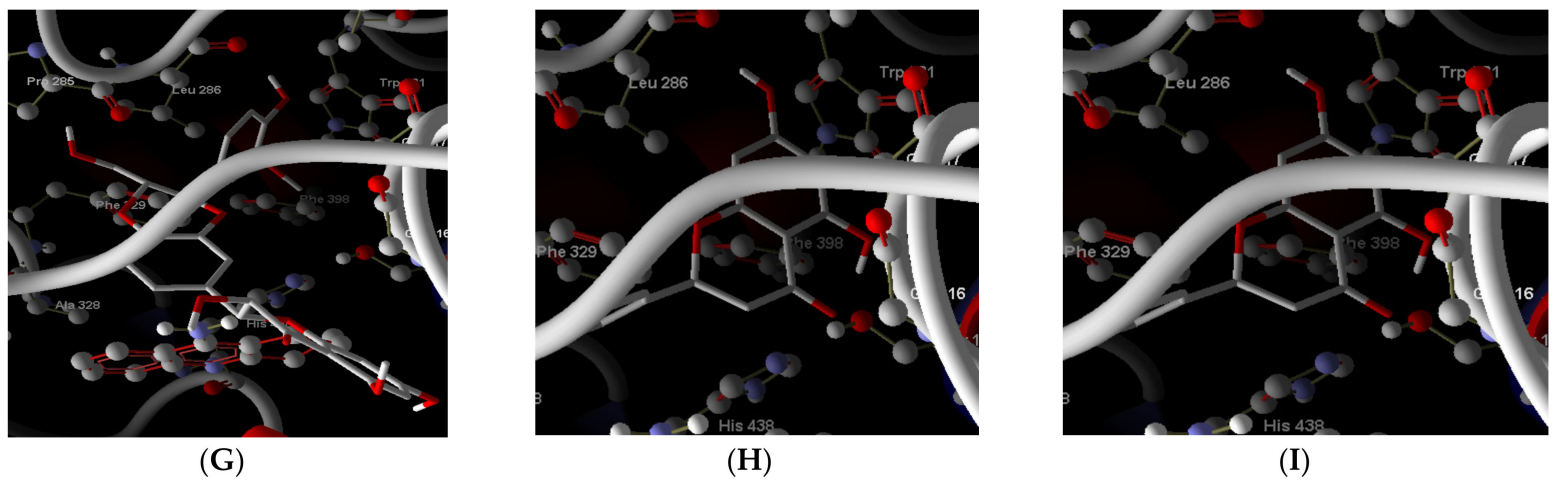
(**A**) Ligands in the workspace of MVD. (**B**) Crystal structure of AChE complex that binds to ligand in the cavity (green). (**C**) Donepezil docked with AChE. (**D**) Silibinin docked with AChE. (**E**) Naringenin docked with AChE. (**F**) Crystal structure of BChE complex that binds to ligand in the cavity (green). (**G**) Rivastigmine docked with BChE. (**H**) Silibinin docked with BChE. (**I**) Naringenin docked with BChE.

ChE inhibitors are often recommended to treat neurodegenerative disorders. Therefore, AChE and BChE inhibitors are now widely used in the treatment and prevention of neurodegenerative diseases. AChE inhibitors have been shown to improve cognition, behaviour, and mood, as well as overall functioning during the course of the disease. In addition, several drugs that may be preventive against the development of AD and Parkinson’s disease are useful treatments against neurogenerative diseases and the NMDA receptor, a partial agonist [42]. For AChE, silibinin shows a MolDock score of −144.245, which is close to the MolDock score of the standard drug donepezil (score of −146.336), while naringenin shows a MolDock score of −120.053. For BChE, silibinin shows a higher affinity towards BChE (MolDock score: −134.604), which is higher than the standard rivastigmine (MolDock score: −114.499), while naringenin shows a MolDock score of −108.614) (Table 6 and Figure 6).

## 4. Discussion

Anthropogenic xenoestrogens such as BPA, when used and disposed of indiscriminately, represent a threat to wildlife, including humans [43]. Human activities are mostly to blame for the steady increases in BPA levels in the environment, including in the air, water, and soil. Neuropsychiatric problems, diabetes, inflammatory reactions, and neurodegeneration have all been associated with BPA exposure [44]. It was necessary in this investigation to use BPA concentrations well above the environmentally relevant level in order to determine the negative effects of increasing the BPA load on zebrafish neurobehavioural responses, anti-oxidant signalling, and neuromorphology. BPA appears to be neurotoxic to zebrafish, as evidenced by our results showing that it causes aberrant neurobehavioural activity, oxidative stress, and neuromorphological abnormalities [45]. BPA’s possible neurotoxic effect is connected to the development of zebrafish neurological symptoms according to our findings. BPA’s neurotoxic potential in zebrafish scototaxis and bottom-dwelling behaviour, as well as the use of silibinin and naringenin as neuroprotective approaches, is significantly supported by these preliminary data. 

*Artemia salina* L. (Artemiidae), a brine-shrimp-like invertebrate that lives in salty aquatic networks, was used in bioassays to determine toxicity by calculating the LC_50_ values, which were recorded for a variety of toxins and plant extracts [46]. A brine shrimp lethality assay has been established in several publications as a simple approach for preliminary toxicity investigations, the screening of medicinal plants, and the monitoring the isolation of diverse bioactive chemicals with LC_50_ values of 1000 μg/mL [47]. In this investigation, the flavonoids silibinin and naringenin had LC_50_ values of >1000 μg/mL, showing wider safety margins than the reference standards.

Acute toxicity testing in zebrafish is widely accepted in aquatic toxicology according to OECD TG 203. The silibinin- and naringenin-treated groups explored the tank’s upper section in the novel tank procedure. Shoal cohesiveness is a term used to describe how zebrafish prefer to swim in clusters. This behaviour is thought to be used by a variety of fish species to evade predators [48]. In the novel tank test procedure, the zebrafish exhibited robust behavioural responses to novelty-evoked anxiety. Such responses are based on the animals’ natural instincts to avoid danger by scuba diving, freezing, and otherwise limiting their exploration of new environments. As the fish adapt to the new surroundings, their exploration tends to grow (e.g., increase in locomotion, increase in the number of entries to the upper level of the tank, decreased freezing). The novel tank paradigm has evolved into a helpful drug screening technique, since anxiogenic and anxiolytic compounds can influence the type of anxious behaviour. For example, zebrafish have been investigated with ethanol, nicotine, morphine, amphetamine, benzodiazepines, and cocaine [49].

Co-supplementation of silibinin and naringenin following waterborne exposure to BPA significantly altered the bottom-dwelling behaviour in zebrafish, compared to naive and control zebrafish. A significant decrease in the number of transitions to the light zone, as well as the amount of time spent in the light zone, was observed in LDPT after co-supplementation with silibinin and naringenin. These preliminary data strongly indicate BPA’s neurotoxic potential in producing zebrafish scototaxis and bottom-dwelling behaviour, as well as the usage of silibinin and naringenin as prospective neuroprotective drugs.

**Figure 7:**
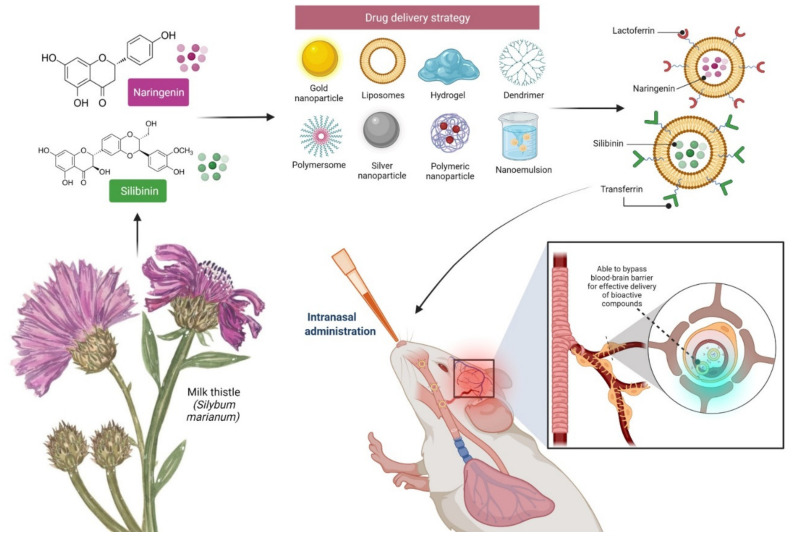
Drug delivery strategy of silibinin and naringenin through the intranasal route. Large compounds addressing developing CNS disorders are unable to reach their target due to the blood–brain barrier (BBB). One of the most promising methods of delivering phytochemicals to the brain is via the use of liposomes functionalised with transferrin (Tf) or lactoferrin (Lf). Transferrin binds to the Tf Receptor (TfR) on brain endothelial cells, while lactoferrin binds to the Lf Receptor (LfR). Both allow bioactive substances such as silibinin and naringenin to cross the BBB via transcytosis, further enhancing their bioavailability and efficacy while reducing toxicity.

Previous studies have shown that silibinin and naringenin have neuroprotective properties in several animal models of CNS illnesses, such as depression, cerebral ischemia, and Alzheimer’s disease, among others, indicating their neurotoxicity’s ameliorative effects and that silibinin therapy reduced A-induced toxic effects on cognitive impairment [50]. It has been observed that silibinin has favourable effects on chronically stressed mice, notably lowering inflammation and oxidative stress [51]. Oral naringenin therapy improved memory deficits in a BPA-induced Alzheimer’s mice model. This antioxidant and anti-inflammatory activities have been demonstrated in numerous studies, showing their role in a number of neurological diseases, in addition to exposing the biological significance in epilepsy, depressive disorders such as depression, Parkinson’s, dementia, and ischemic strokes [52]. Taken together, the findings suggest that neuroprotective flavonoids may have a wide range of therapeutic applications, requiring further research into their molecular mechanisms, drug delivery strategy, and safety profile (Figure 7).

In the current investigation, the neuropharmacological activity of silibinin and naringenin on the neuroprotective efficacy induced by bisphenol A has been confirmed, as evidenced by the behavioural parameters assessed after the 21 day studies. Because of their genetic and physiological parallels to the human system, mice have been chosen as the best model for neuroprotective action. Due to their simplicity and capacity to detect different and common behavioural alterations with bisphenol A, behavioural measures are useful for neuroprotective assessments in zebrafish. More research is needed to determine the toxicity and long-term effects of silibinin and naringenin on anxiety disorders. 

Silibinin and naringenin demonstrated compelling and well-commensurate results to standard drugs in molecular docking experiments for both AD and PD. BPA-induced neurotoxicity degrades the glutathione pathway by downregulating cellular antioxidants such as various antioxidant-scavenging enzymes, including GSH. In the zebrafish model, oxidative stress is directly associated with changes in neurobehavioural responses and neurodegeneration, which is exacerbated by further waterborne exposure to BPA. As a result, co-supplementation of silibinin and naringenin provides considerable neuroprotection against BPA-induced oxidative stress and neuronal damage as a preventative strategy. Forthcoming investigations aimed at unravelling the downstream signalling pathways may provide inputs for creating prophylactic strategies against BPA-induced predilection in the development of significant neurological disorders. It is envisaged that the research will play a key role in lowering senile behaviours among the elderly, which will benefit not only society, but also the nation and the world as a whole.

Further research to elucidate the mechanisms of silibinin and naringenin in neuro-protective development can yield new knowledge of drug development for neurodegenerative disorders. The determination of the toxicity and long-term effects of silibinin and naringenin when validating a zebrafish model for the neuroprotective impacts of bisphenol A, an anthropogenic xenoestrogen, will also be useful. Additionally, biochemical studies can support silibinin and naringenin’s potential therapeutic efficacy against BPA-induced oxidative stress in zebrafish. Long-term pharmacological research and pharmacokinetic studies of silibinin and naringenin are also essential, together with the identification of molecular markers of BPA-induced neurotoxicity and epigenetic changes generated by silibinin and naringenin treatments.

## 5. Conclusions

Silibinin and naringenin are invigorate flavonoids that ameliorate oxidative stress, neuroinflammation, and neuro-apoptotic effects, resulting in better cognitive performance. The in silico study on silibinin and naringenin showed their potential neuroprotective properties due to their ability to bind to specific targets of AD and PD. Overall, the findings of our research suggest that silibinin and naringenin could be useful in the development of novel drugs to treat neurodegenerative diseases.

## Figures and Tables

**Table 1:** Acute toxicity test conditions for the limit test of OECD TG 203.

Test Substance	Silibinin and Naringenin
Test fish	Zebrafish (*Danio rerio*); Strain: wild-type, 4–6 months old
Test procedure	Static method
pH	6.0 to 8.5
Temperature	21–25 °C
Control	A group exposed to reverse osmosis water serves as control
Test concentration	Silibinin/Naringenin (100 mg/L) for performing the limit test
Number of fish	10 fish per concentration for the test sample and control
Observation	Fish behaviour was closely observed and the number of deaths was also recorded at intervals of 24, 48, 72, and 96 h.

**Table 2:** Experimental design for neuropharmacological evaluation.

Environmental Conditions	Temperature Was Maintained in the Range of 26 ± 1.5 °C, pH 7–8, Dissolved Oxygen Concentration of not less than 60%.
Housing	Zebrafish were housed in groups in a 40 L tank with dechlorinated water with constant aeration and filtration.
Diet & Water	Provided with commercially available flake food with Artemia salina (brine shrimp) nauplii and dechlorinated water.
Feeding	Brine shrimp nauplii provided once daily and flake food twice daily for one day before the start of the study
Test drug	Silibinin and naringenin. Test solutions freshly prepared prior to administration.
Inducing agent	Bisphenol A
Control	A control group was maintained
Concentration of test drug	10 µM
Concentration of inducing agent	17.52 µM
Treatment	For 21 consecutive days by tank water immersion
Evaluation	Novel tank test: on day 22 Light/dark test: on day 22

**Table 3:** Brine shrimp lethality assay for silibinin and naringenin.

S.no	Concentration (µg/mL)	Percentage Death of Nauplii following 24 h of Exposure		
		Silibinin	Naringenin	Potassium Dichromate
1	0.1	21.68 ± 1.66	19.00 ± 2.64	25.00 ± 2.88
2	1.0	26.66 ± 1.45	30.34 ± 2.72	36.35 ± 1.85
3	10.0	33.33 ± 2.33	44.00 ± 1.52	59.33 ± 1.66
4	100.0	40.66 ± 1.66	52.23 ± 1.34	78.65 ± 1.46
5	1000.0	54.00 ± 1.54	77.66 ± 1.60	99.00 ± 1.15

**Table 4:** LC_50_ values for silibinin, naringenin, and potassium dichromate.

Log (Inhibitor) vs. Response			
Best-Fit Values	Silibinin	Naringenin	Potassium Dichromate
Bottom	80.11	53.00	94.00
Top	28.66	26.03	29.21
LogLC_50_	1.959	1.534	1.219
LC_50_	91.34	35.10	13.15
Span	−52.32	−26.96	−64.79

**Table 5:** Observation of mortality and abnormalities in the acute toxicity test (96 h).

Compound	Exposure Concentration	Mortality				Abnormal Changes				
		24 h	48 h	72 h	96 h	Swimming	LORF	LOE	Pigmentation	Others
						Signs				
Blank	-	-	-	-	-	-	-	-	-	-
Control (reverse osmosis water)	-	-	-	-	-	-	-	-	-	-
Silibinin	100 mg/L	-	-	-	-	-	-	-	-	-
Naringenin	100 mg/L	-	-	-	-	-	-	-	-	-

LORF: Loss of respiratory function; LOE: loss of equilibrium.

**Table 6:** Comparative analysis of ligand interactions with silibinin and naringenin with cholinesterase receptors.

Compound	Acetylcholinesterase			Butyrylcholinesterase		
	MolDock Score	Rerank Score	HBond	MolDock Score	Rerank Score	HBond
Donepezil	−146.449	−115.75	0	-	-	-
Rivastigmine	-	-	-	−114.933	−82.351	−1.2642
Silibinin	−164.255	−17.442	−12.855	−156.414	−39.779	−10.363
Naringenin	−126.023	−17.442	−12.855	−114.323	−89.786	−9.9648

## Data Availability

The data presented in this study are available upon request from the corresponding author.
